# Aberrant multimodal brain networks in patients with anti‐NMDA receptor encephalitis

**DOI:** 10.1111/cns.13632

**Published:** 2021-03-13

**Authors:** Jinhui Wang, Yunyun Duan, Tian Zhang, Jing Huang, Zhuoqiong Ren, Jing Ye, Ningkai Wang, Yinzhi Li, Xiaoya Chen, Peiyi Gao, Kuncheng Li, Yaou Liu

**Affiliations:** ^1^ Institute for Brain Research and Rehabilitation Guangdong Key Laboratory of Mental Health and Cognitive Science Center for Studies of Psychological Application South China Normal University Guangzhou China; ^2^ Key Laboratory of Brain, Cognition and Education Sciences (South China Normal University) Ministry of Education Guangzhou China; ^3^ Department of Radiology Beijing Tiantan Hospital Capital Medical University Beijing China; ^4^ Department of Radiology Xuanwu Hospital Capital Medical University Beijing China; ^5^ Department of Radiology The First Affiliated Hospital of Chongqing Medical University Chongqing China; ^6^ Tiantan Image Research Center China National Clinical Research Center for Neurological Diseases Beijing China

**Keywords:** anti‐NMDAR encephalitis, brain network, cognitive impairment, lateral orbital gyrus, multimodal magnetic resonance imaging

## Abstract

**Aims:**

To explore large‐scale brain network alterations and examine their clinical and neuropsychological relevance in patients with anti‐N‐methyl‐D‐aspartate receptor (NMDAR) encephalitis.

**Methods:**

Twenty‐four patients with anti‐NMDAR encephalitis and 26 matched healthy controls (HCs) were enrolled in our study. Based on the multimodal MRI dataset, individual morphological, structural, and functional brain networks were constructed and compared between the two groups at multiple levels. The associations with clinical/neuropsychological variables and the discriminant ability of significant alterations were further studied.

**Results:**

Multimodal network analysis revealed that anti‐NMDAR encephalitis mainly affected morphological and structural networks, but subtle alterations were observed in functional networks. Intriguingly, decreased network local efficiency was observed for both morphological and structural networks and increased nodal centrality in the lateral orbital gyrus was convergently observed among the three types of networks in the patients. Moreover, the alterations, particularly those from structural networks, accounted largely for cognitive deficits of the patients and could distinguish the diseased individuals from the HCs with excellent performance (area under the curve =0.933).

**Conclusions:**

The current study provides a comprehensive view of characteristic multimodal network dysfunction in anti‐NMDAR encephalitis, which is crucial to establish new diagnostic biomarkers and promising therapeutic targets for the disease.

## Graphical abstract

The current study provides a comprehensive view of characteristic multimodal network dysfunction in anti‐N‐methyl‐D‐aspartate receptor encephalitis, which is crucial to establish new diagnostic biomarkers and promising therapeutic targets for the disease.

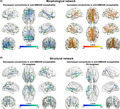

## 1 INTRODUCTION

Anti‐N‐methyl‐D‐aspartate receptor (anti‐NMDAR) encephalitis was first described by Dalmau in 2007 ^1^ as a common type of autoimmune‐mediated limbic encephalitis with auto‐antibodies that targets neuronal surface or synaptic antigens. Patients with anti‐NMDAR encephalitis present various clinical symptoms, including behavioral and psychiatric features, memory and cognitive deficits, seizures, central hypoventilation, and movement disorders.^2^, ^3^, ^4^, ^5^

Although 33%–55% patients with anti‐NMDAR encephalitis show brain lesions (e.g., medial temporal, frontal, and parietal cortex), normal brain images are observed in the majority of patients with routine clinical MRI sequences.^4^ In contrast, advanced multimodal MRI techniques can successfully detect brain alterations in anti‐NMDAR encephalitis, such as morphological atrophy,^6^ disrupted white‐matter microstructure ^7^ and altered cerebral blood flow.^8^ Beyond the focal alterations, recent studies have demonstrated that anti‐NMDAR encephalitis is associated with impaired interregional connectivity not only in single neural circuit ^7^ but spanning multiple distributed brain systems,^9^ implying that anti‐NMDAR encephalitis is a global network dysfunctional disease.

Previous studies have indicated that large‐scale human brain networks can be mapped from multimodal MRI data and further characterized by graph‐based network approaches. It has been well documented that human brain networks possess many non‐trivial topological features, including efficient small‐worldness, modularity, and hubs.^10^ Moreover, mounting evidence suggests that various brain disorders are associated with specific disturbances in these configurations.^11^ Regarding anti‐NMDAR encephalitis, however, it remains elusive whether and how large‐scale brain networks are disrupted and to what extent the disruptions (if observed) could account for cognitive dysfunction, of the patients.

In this study, we aimed to provide a comprehensive view of topological alterations of large‐scale brain networks in anti‐NMDAR encephalitis. To achieve this goal, we constructed and compared morphological, structural, and functional networks in 24 patients with anti‐NMDAR encephalitis versus 26 healthy controls (HCs) by combining anatomical, diffusion, and resting‐state functional MRI. The combination of multimodal MRI is vital since different modalities are complementary in revealing the network organization of the human brain.^12^, ^13^ Specifically, we set out to assess (i) common and specific alterations among multimodal brain networks in anti‐NMDAR encephalitis at multiple levels and (ii) cognitive relevance of the network alterations via examination of their associations with neuropsychological variables of the patients and their discriminative power in distinguishing patients from HCs.

## 2 METHODS

### 2.1 Participants

Twenty‐seven patients with anti‐NMDAR encephalitis and 27 age‐, sex‐, and education‐matched HCs were enrolled in our study. All the patients showed positive anti‐NMDAR antibodies in the cerebrospinal fluid (CSF) samples according to previously reported criteria.^2^, ^4^ Three patients and one control were excluded from further analysis due to excessive head motion during the functional MRI scan. This study was approved by the institutional review board of Xuanwu hospital, Capital Medical University, Beijing, China, and written informed consent was obtained from each participant (Supporting Information S1).

### 2.2 Neuropsychological assessment

A comprehensive cognitive test battery was administered to each participant by a trained investigator who was blinded to the clinical and MRI results of the participants. The assessment included the Paced Auditory Serial Addition Task (PASAT; accessing auditory processing speed, attention, and working memory), the Symbol Digit Modalities Test (SDMT; accessing the visual processing speed and working memory), the California Verbal Learning Test—Second Edition (CVLT; accessing the auditory or verbal episodic memory), the Brief Visuospatial Memory Test—Revised (BVMT, accessing the visual or spatial episodic memory), and the Mini‐Mental State Examination (MMSE).

### 2.3 Multimodal MRI data acquisition

Each participant underwent multimodal MRI scanning (T2‐weighted, anatomical, diffusion, and resting‐state functional) on a 3.0 Tesla MR system (Siemens Magnetom Trio Tim system) in Xuanwu hospital, Capital Medical University (Supporting Information S1).

### 2.4 Multimodal MRI data preprocessing

Before constructing brain networks, the multimodal MRI data underwent a series of modality‐dependent preprocessing steps.

#### 2.4.1 Anatomical MRI data

The anatomical MRI data were processed using the VBM8 toolbox (http://dbm.neuro.unijena.de/vbm8) for SPM8 (http://www.fil.ion.ucl.ac.uk/spm/software/spm8/). Briefly, individual images were first segmented into gray matter (GM), WM, and CSF using an adaptive Maximum A Posterior technique. The resultant GM maps were then normalized to the standard Montreal Neurological Institute (MNI) space using a high‐dimensional “DARTEL” approach and were non‐linearly modulated to compensate for spatial normalization effects. The modulated GM maps were further spatially smoothed using a Gaussian kernel with 6‐mm full width at half maximum.

#### 2.4.2 Diffusion‐weighted MRI data

The diffusion‐weighted MRI data were processed using the PANDA toolbox^14^ based on FMRIB Software Library (FSL) (https://fsl.fmrib.ox.ac.uk/fsl/fslwiki/). First, individual diffusion‐weighted images were co‐registered to their corresponding b0 images using an affine transformation to correct for head motion and eddy current‐induced distortions. Then, a diffusion tensor model was estimated at each voxel and diagonalization was performed to yield three eigenvalues and eigenvectors.^15^

#### 2.4.3 Resting‐state functional MRI data

The resting‐state functional MRI data were preprocessed using the GRETNA toolbox^16^ based on SPM8 (http://www.fil.ion.ucl.ac.uk/spm/software/spm8/). After discarding the first five volumes, individual functional images were corrected for intra‐volume temporal offsets (Sinc interpolation) and inter‐volume head motion (rigid‐body transformation). Four participants (3 patients and 1 control) were excluded from further analysis in terms of the criterion of a displacement >3 mm, an angular rotation >3°, or a mean frame‐wise displacement >0.5. The corrected images were then spatially normalized into the MNI space via transformation fields derived from tissue segment of individual T1 images. The resultant normalized images further underwent removal of linear trend and temporal band‐pass filtering (0.01–0.08 Hz). Finally, several nuisance signals, including 24‐parameter head motion profiles,^17^ WM signals, and CSF signals were regressed out from each voxel's time series to exclude non‐neuronal sources. The WM and CSF signals were obtained by separately averaging time series within the WM and CSF brain masks derived from prior probability maps in the SPM8 toolbox (threshold = 0.8) for each individual.

### 2.5 Multimodal brain network construction

After modality‐dependent preprocessing, we constructed large‐scale morphological, structural (fractional anisotropy [FA] weighted, fiber number [FN] weighted and fiber length [FL] weighted], and functional networks for each participant based on anatomical, diffusion, and resting‐state functional MRI data, respectively. In these networks, nodes represented brain regions in terms of the automated anatomical labeling atlas (47 regions in each hemisphere)^18^, ^19^ and edges represented interregional morphological similarity,^20^, ^21^ fiber pathway, or functional connectivity depending on the imaging modalities. For each network, we then calculated multiple global (local efficiency, global efficiency and modularity, as well as their normalized versions by random networks) and nodal (degree, efficiency, betweenness, eigenvector, and pagerank) network measures (Supporting Information S1).

### 2.6 Cross‐modality relationship among multimodal brain networks

In this study, we constructed individual morphological, structural, and functional brain networks. Previous studies have shown that although different types of brain networks resemble and interrelate with each other, there are no determinant even poor correspondences between them.^22^, ^23^, ^24^ Moreover, it is frequently reported that structure‐function network coupling is disrupted in brain disorders, and the disruption is related to clinical severity of patients, such as depression, schizophrenia, and epilepsy.^25^, ^26^, ^27^ Thus, we also examined whether cross‐modality relationships were disrupted in patients with anti‐NMDAR encephalitis. Specifically, we calculated pairwise Spearman correlation coefficients among morphological, structural (FA, FN, and FL weighted), and functional brain networks with respect to their connectivity patterns (across connections) and nodal centrality profiles (across nodes). This resulted in, for each participant, 10 correlation coefficients for the connectivity patterns and 10 correlation coefficients for each nodal centrality measure. Notably, for the cross‐modality correlations of connectivity patterns, only connections that existed (i.e., non‐zero) in individual structural brain networks were used. Based on previous findings, we expect that significant positive correlations will be found for the cross‐modality network relationships and that the cross‐modality relationships will be disrupted in the disease.

### 2.7 Statistical analysis

All continuous variables (demographic data, neuropsychological tests, multimodal network measures, and cross‐modality relationships) were tested for normality with Lilliefors tests. Parametric two‐sided two‐sample *t*‐tests or nonparametric Wilcoxon rank‐sum tests were used for continuous demographic and neuropsychological variables depending on whether they were normally distributed. Sex data were examined with a chi‐square test. Since nearly all network measures and cross‐modality relationships were not normally distributed, nonparametric permutation tests (10,000 permutations) were used to statistically infer their between‐group differences. For interregional connectivity, a network‐based statistic (NBS) method^28^ was used to examine between‐group differences. For measures showing significant alterations in the patients, we further examined their associations with clinical and neuropsychological variables (disease duration, MMSE, SDMT, CVLT, BVMT, and PASAT) of the patients (Pearson or Spearman correlation) and their discriminant power in distinguishing the patients from HCs (receiver operating characteristic curve analysis). A false discovery rate (FDR) procedure was used to correct for multiple comparisons at a *q* value of 0.05 for between‐group comparisons of nodal measures and cross‐modality relationships, MRI‐clinical/neuropsychological correlation analyses, and the receiver operating characteristic curve analyses (Supporting Information S1).

### 2.8 Post hoc examination of susceptibility artifact signal loss

Given that the right lateral orbital gyrus, a region prone to diffusion and functional MRI signal loss, was consistently observed to show centrality alterations in the patients, we examined possible confounding effect of the signal loss on our results (Supporting Information S1).

### 2.9 Validation analysis using functional parcellation atlases

How to define network nodes is an ongoing topic of research for brain network studies, and there is no consensus on the optimal choice for defining brain network nodes.^29^ Generally speaking, anatomical parcellation atlases are typically suitable for structural brain network studies while functionally defined regions are suitable for functional brain network studies. According to these rules, however, it is difficult to choose a suitable way to define network nodes for this study since this work was a multimodal study that combined morphological, structural, and functional brain networks. Here, we selected the anatomical AAL atlas because it is one of the mostly used atlases in previous brain networks studies. More importantly, it has been utilized to study brain networks in patients with anti‐NMDAR encephalitis.^9^ Thus, utilizing the AAL atlas allows direct comparisons between our results and previous findings. To test whether our functional findings are reproducible, we further constructed individual functional brain networks using functionally defined atlases.^30^ The atlases are derived from resting‐state fMRI data from 1489 subjects using a gradient weighted markov random field approach, and divide the brain into 100, 200, and 400 regions, respectively.

## 3 RESULTS

### 3.1 Demographic, clinical, and cognitive characteristics

No significant differences were found between the two groups in age or sex (*p* > 0.05). However, the patients had slightly less years of education than the HCs (*p* = 0.048). Compared with the HCs, the patients performed worse in all cognitive tests, including the PASAT‐2 seconds (*p* < 0.001), PASAT‐3 seconds (*p* = 0.002), CVLT (*p* = 0.002), BVMT (*p* = 0.002), SDMT (*p* < 0.001), and MMSE (*p* = 0.003) (Table 1).

**TABLE 1:** Demographic, clinical, and neuropsychological data for all participants

	HCs (*n* = 26)	Anti‐NMDAR encephalitis (*n* = 24)	*p* value
Age (years)	27.4 (8.3)	28.4 (12.0)	0.734
Sex (M/F)	14/12	14/10	0.749
Education (years)	15 (9–20)	13 (9–17)	0.048
Duration (months)		18.2 (9.4)	
Number of relapse		0 (0–4)	
mRS		0 (0–2)	
MMSE^a^	29 (27–30)	28 (21–30)	0.003
SDMT^a^	66.870 (9.560)	49.375 (12.430)	<0.001
CVLT^a^	54.652 (8.695)	43.625 (13.641)	0.002
BVMT^a^	29.304 (4.139)	22.750 (8.594)	0.002
PASAT−3 seconds^b^	58 (45–60)	42 (18–60)	0.002
PASAT−2 seconds^b^	45.348 (7.625)	33.235 (12.194)	<0.001

Note

For continuous variables, data are represented as mean (SD) or median (minimum–maximum) depending on whether they are normally distributed.

Abbreviations: BVMT, Brief Visuospatial Memory Test; CVLT, California Verbal Learning Test; HCs, healthy controls;MMSE, Mini‐Mental State Examination; mRS, modified Rankin Scale; NMDAR, N‐methyl‐D‐aspartate receptor; PASAT, Paced Auditory Serial Addition Test; SDMT, Symbol Digit Modalities Test.

^a^ Data are missing for three healthy controls.

^b^ Data are missing for three healthy controls and seven patients.

### 3.2 Altered global network measures in the patients

**FIGURE 1:**
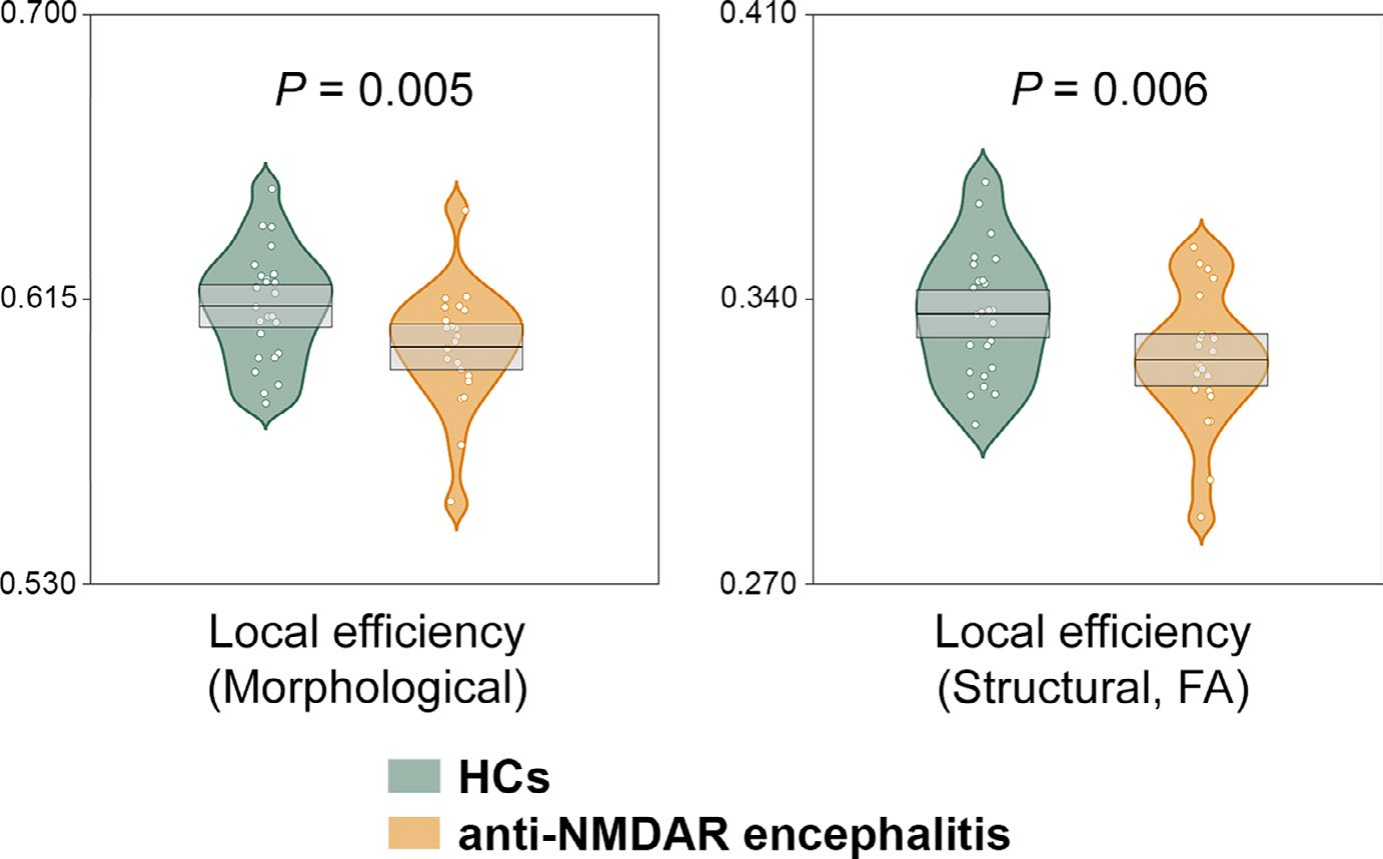
Decreased local network efficiency in the patients. HCs, healthy controls; FA, fractional anisotropy; NMDAR, N‐methyl‐D‐aspartate receptor

Global network alterations are summarized in Figure 1.

#### 3.2.1 Morphological networks

Compared with the HCs, the patients exhibited significantly decreased local efficiency (*p* = 0.005).

#### 3.2.2 Structural networks

(a) FA weighted networks. Compared with the HCs, the patients exhibited significantly decreased local efficiency (*p* = 0.006). (b) FN weighted networks. No significant alterations were observed in the patients (*p* > 0.05, Bonferroni corrected). (c) FL weighted networks. No significant alterations were observed in the patients (*p* > 0.05, Bonferroni corrected).

#### 3.2.3 Functional networks

No significant alterations were observed in the patients (*p* > 0.05, Bonferroni corrected).

### 3.3 Altered nodal network measures in the patients

**FIGURE 2:**
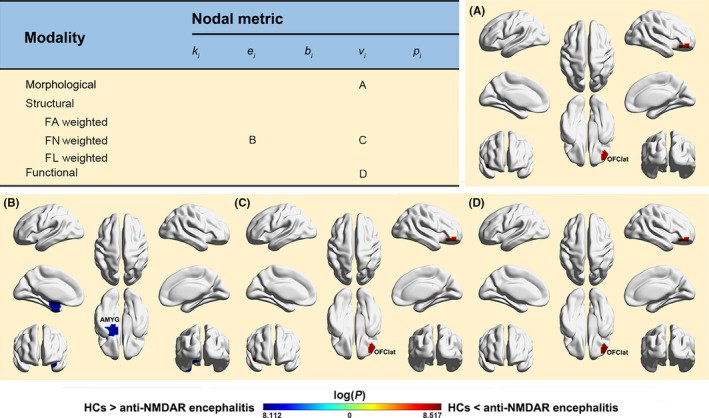
Altered nodal centralities in the patients. FA, fractional anisotropy; FN, fiber number; FL, fiber length; OFClat, lateral orbital gyrus; AMYG, amygdala; *k*_i_, nodal degree; *e*_i_, nodal efficiency; *b*_i_, nodal betweenness; *v*_i_, nodal eigenvector; *p*_i_, nodal pagerank

Nodal network alterations are summarized in Figure 2.

#### 3.3.1 Morphological networks

Compared with the HCs, the patients showed significantly increased nodal eigenvector in the right lateral orbital gyrus (*p* < 0.05, FDR corrected).

#### 3.3.2 Structural networks

(a) FA weighted networks. No significant alterations were observed in the patients (*p* > 0.05, FDR corrected). (b) FN weighted networks. Compared with the HCs, the patients showed significantly decreased nodal efficiency in the left amygdala and increased nodal eigenvector in the right lateral orbital gyrus (*p* < 0.05, FDR corrected). (c) FL weighted networks. No significant alterations were observed in the patients (*p* > 0.05, FDR corrected).

#### 3.3.3 Functional networks

Compared with the HCs, the patients showed significantly increased nodal eigenvector in the right lateral orbital gyrus (*p* < 0.05, FDR corrected).

### 3.4 Altered interregional connectivity in the patients

**FIGURE 3:**
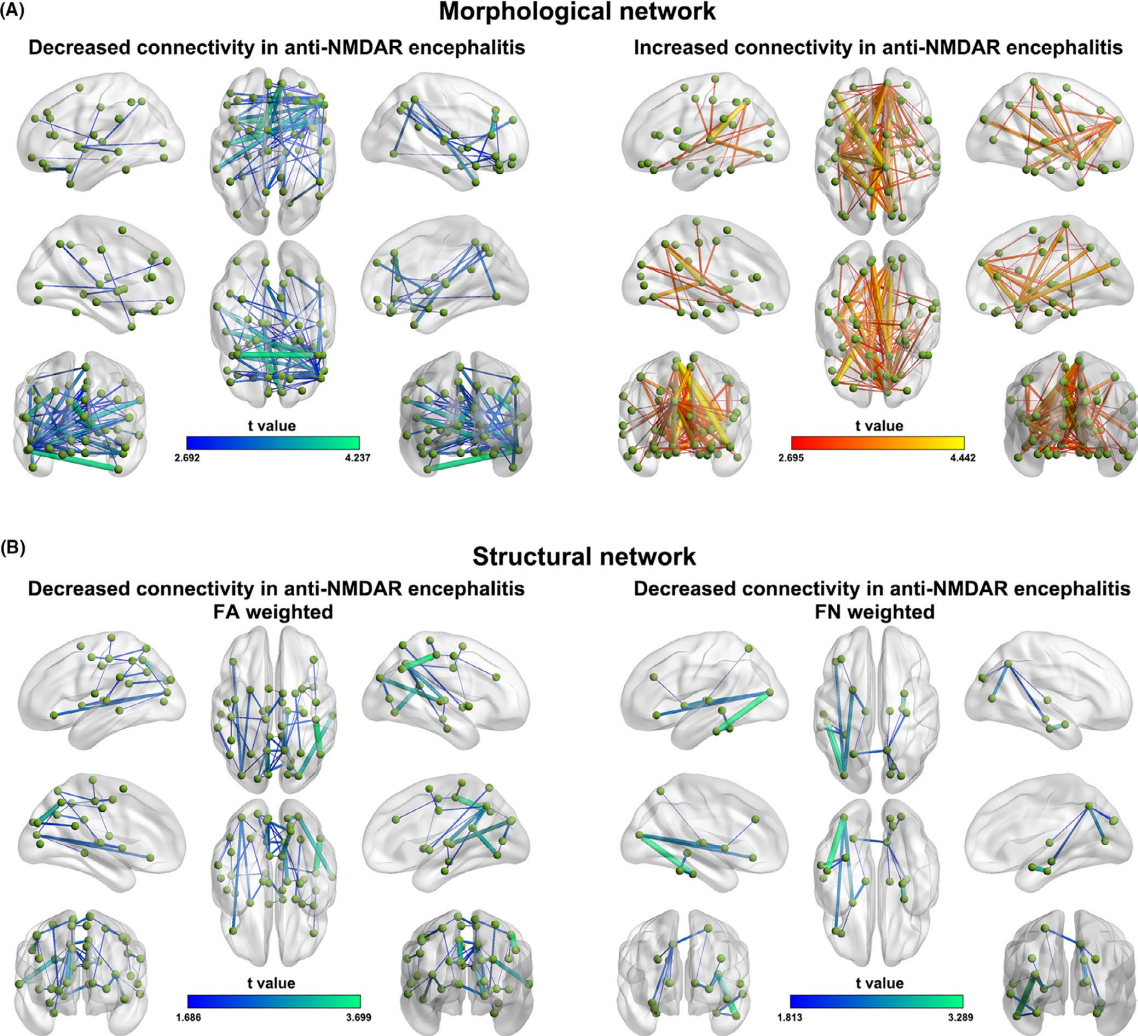
Altered morphological (A) and structural (B) connectivity in the patients. FA, fractional anisotropy; FN, fiber number; NMDAR, N‐methyl‐D‐aspartate receptor

Interregional connectivity alterations are summarized in Figure 3.

#### 3.4.1 Morphological networks

Compared with the HCs, two components were identified to show altered connectivity in the patients (Figure 3A): One exhibited decreased connectivity (*p* = 0.019), and the other showed increased connectivity (*p* = 0.002). The decreased component included 70 connections linking 49 regions that were primary short‐range (< 75 mm) edges (40, 57.1%) and association cortical and limbic/paralimbic regions (41, 83.7%), while the increased component included 107 connections linking 59 regions that were also primary short‐range edges (56, 52.3%) and association cortical and limbic/paralimbic regions (45, 76.3%).

#### 3.4.2 Structural networks

(a) FA weighted networks. Compared with the HCs, a single component was identified to show significantly decreased connectivity in the patients (*p* = 0.005) (Figure 3B, left). The component included 51 connections linking 42 regions that were primarily short‐range edges (46, 90.2%) and association cortical regions (23, 54.8%). (b) FN weighted networks. A single component was observed to show decreased connectivity in the patients compared with the HCs (*p* = 0.023) (Figure 3B, right). The component included 17 connections linking 18 regions that were predominantly short‐range edges (14, 82.4%) and association cortices (10, 55.6%). (c) FL weighted networks. No significant alterations were observed in the patients.

#### 3.4.3 Functional networks

No significant alterations were observed in the patients.

### 3.5 Cross‐modality relationships

**FIGURE 4:**
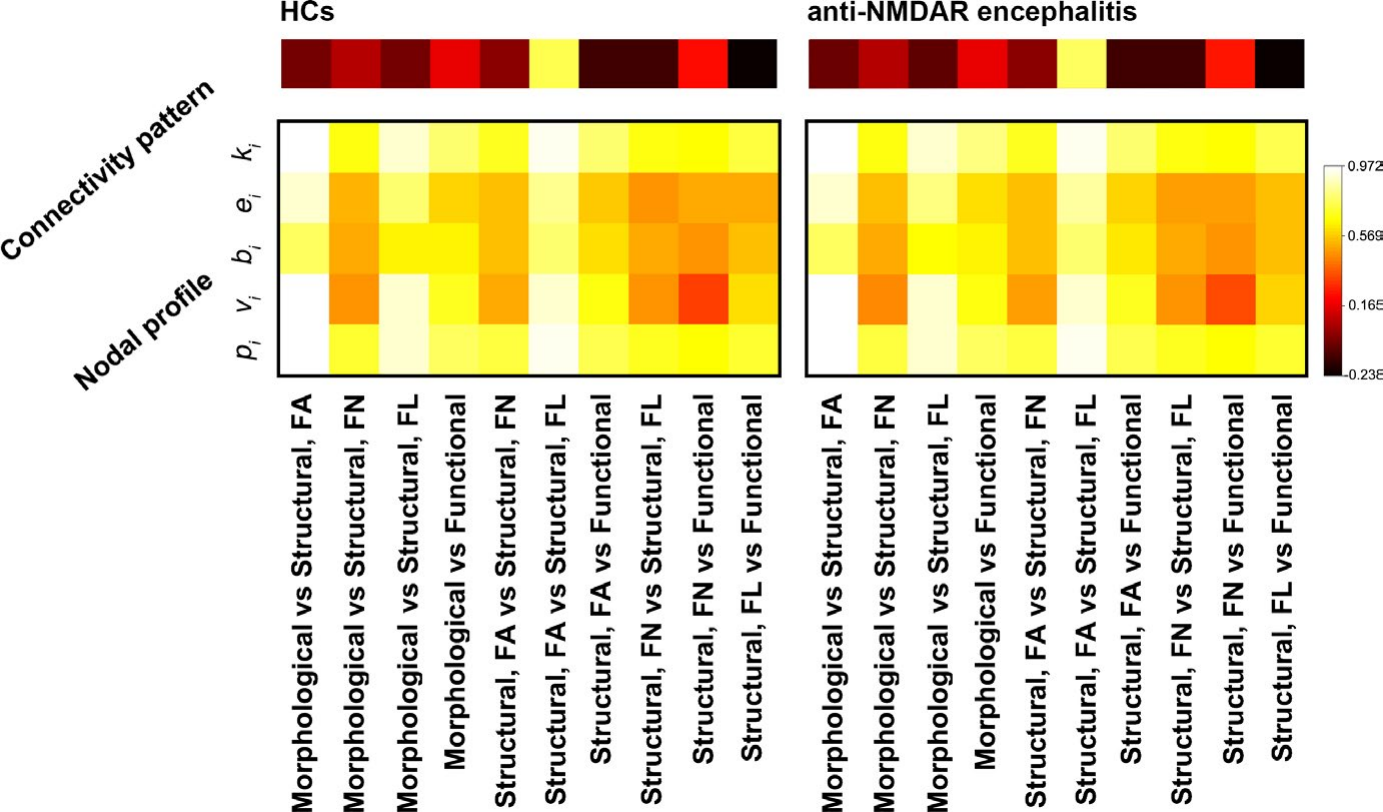
Cross‐modality relationships. No significant differences were observed in cross‐modality relationships of neither connectivity patterns nor nodal centrality profiles in the patients. HCs, healthy controls; NMDAR, N‐methyl‐D‐aspartate receptor; FA, fractional anisotropy; FN, fiber number; FL, fiber length; *k*_i_, nodal degree; *e*_i_, nodal efficiency; *b*_i_, nodal betweenness; *v*_i_, nodal eigenvector; *p*_i_, nodal pagerank

As expect, highly positive correlations were found between different types of brain networks in nodal centrality profiles (Figure 4). While low correlations were observed for the whole‐brain connectivity patterns. Further between‐group comparisons revealed no significant differences in any cross‐modality relationship in either whole‐brain connectivity patterns or nodal centrality profiles (*p* > 0.05, FDR corrected) (Figure 4).

### 3.6 Relationships between network measures and neuropsychological variables

**FIGURE 5:**
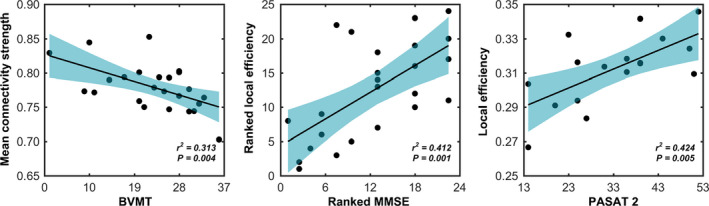
Brain‐neuropsychological associations. Pearson or Spearman correlation was used to examine relationship between multimodal brain network measures and neuropsychological variables in the patients. The results were corrected for multiple comparisons with a false discovery rate procedure. BVMT, Brief Visuospatial Memory Test; MMSE, Mini‐Mental State Examination; PASAT, Paced Auditory Serial Addition Test

Significant correlations were observed between multimodal network measures and neuropsychological variables in the patients (*p* < 0.05, FDR corrected) (Figure 5).

#### 3.6.1 Morphological networks

A significantly negative correlation was observed between mean strength of the increased NBS component and BVMT in the patients (*r* ^2^ = 0.313, *p* = 0.004).

#### 3.6.2 Structural networks

(a) FA weighted networks. Significantly positive correlations were observed between local efficiency and MMSE (*r* ^2^ = 0.412, *p* = 0.001) and PASAT 2 (*r* ^2^ = 0.424, *p* = 0.005) in the patients. (b) FN weighted networks. No significant correlations were observed in the patients (*p* > 0.05, FDR corrected).

#### 3.6.3 Functional networks

No significant correlations were observed (*p* > 0.05, FDR corrected).

### 3.7 Discriminant results

**FIGURE 6:**
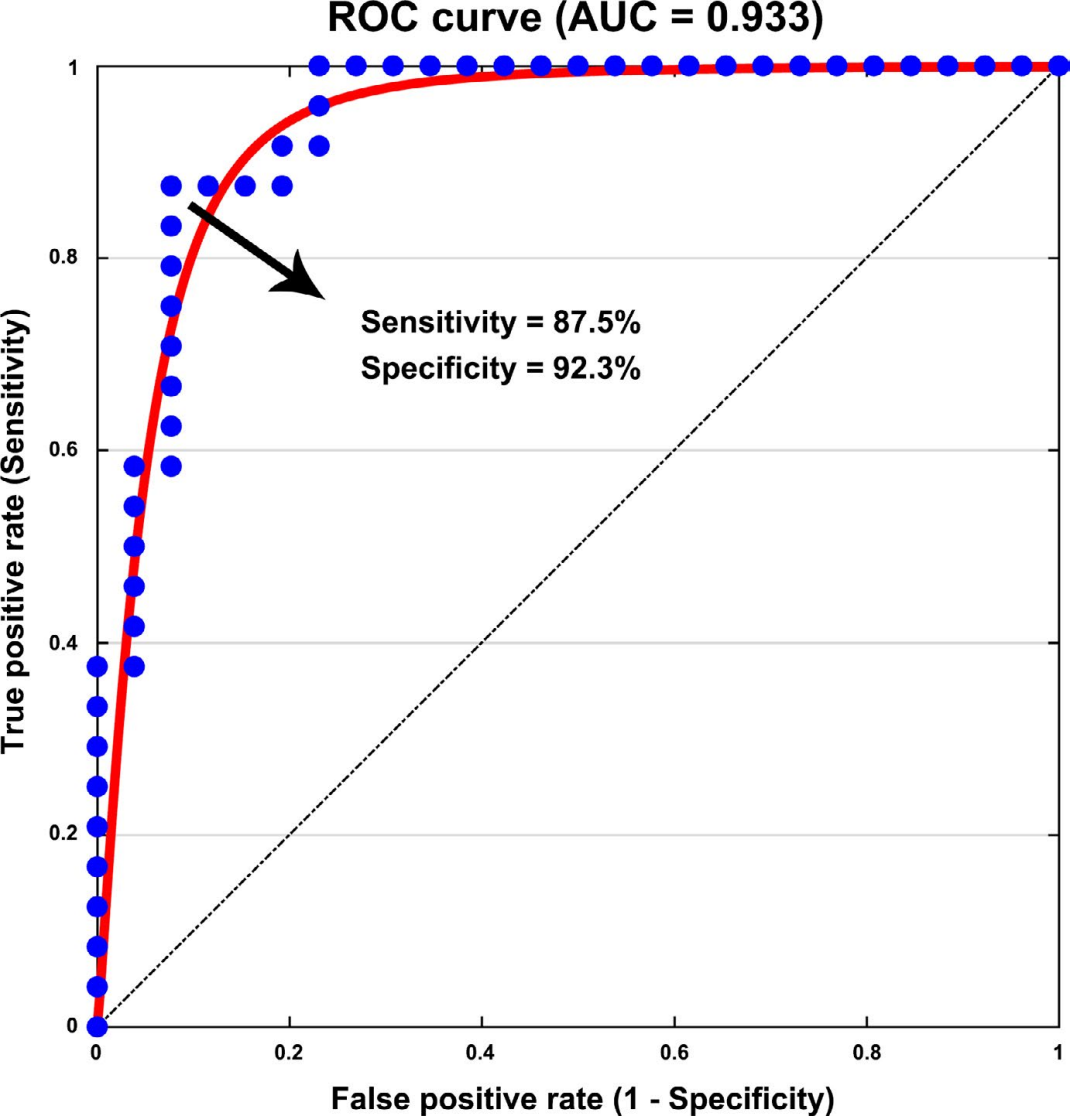
Brain network‐based classification of the patients. The mean connectivity strength of altered connections in the FA‐weighted structural brain networks exhibited high sensitivity and specificity for distinguishing the patients from controls. AUC, area under the curve

Mean strength in the NBS component of the FA‐weighted structural networks exhibited the highest power for distinguishing the patients from the HCs (area under the curve = 0.933, *p*_permutation_ < 0.001, *p*_bootstrap_ *= *0.420), with a sensitivity of 87.5% and a specificity of 92.3% (Figure 6). As such, 21 out of the 24 patients with anti‐NMDAR encephalitis and 24 out of the 26 HCs were classified correctly, generating an accuracy = 90%. Additionally, the mean strength in the NBS components (increased and decreased) of the morphological networks and mean strength in the NBS component (decreased) of the FN‐weighted structural networks exhibited excellent discriminative power (area under the curve = 0.882, 0.843 and 0.882, respectively; all *p*_permutation_ < 0.001 and *p*_bootstrap_ > 0.05). All other network metrics exhibited lower discriminative performance (area under the curve < 0.8).

### 3.8 Effect of susceptibility artifact in the right lateral orbital gyrus

The right lateral orbital gyrus had comparable signals between the two groups for the functional MRI data (*p* > 0.05) but exhibited lower signals for the diffusion MRI data in the patients (*p* = 0.009), which had little effect on our findings.

### 3.9 Validation results

When using functionally defined atlases to construct individual functional brain networks, no significant between‐group differences were found in any interregional functional connectivity, global network attribute or nodal centrality measure regardless of the atlas resolution (*p* > 0.05, corrected). These findings are largely consistent with those derived from the AAL atlas, which collectively suggest that topological architecture of functional brain networks might be intact in patients with anti‐NMDAR encephalitis, although further studies are required by employing other functional atlases.

## 4 DISCUSSION

Combining multimodal MRI and graph‐based network approaches, this study systematically investigated topological alterations of morphological, structural, and functional networks in anti‐NMDAR encephalitis. We found that anti‐NMDAR encephalitis mainly affected morphological and structural brain networks, but subtle alterations were observed in functional brain networks. Moreover, the alterations especially in structural brain networks correlated well with cognitive impairments of the patients and showed excellent performance for differentiating diseased individuals from HCs. These findings offer novel insights into network dysfunction in anti‐NMDAR encephalitis, which contributes to cognitive disturbances in the disease.

Our neuropsychological analysis revealed that the patients with anti‐NMDAR encephalitis showed lower scores in all cognitive tests (i.e., PASAT, BVMT, SDMT, CVLT, and MMSE) compared with the HCs, implying cognitive impairments in multiple domains in the patients, including verbal memory, spatial episodic memory, and information processing speed. Our findings are consistent with previous studies,^31^, ^32^ together confirming pivotal role of NMDARs for executive functions and memory.^33^

For multimodal brain networks, previous studies have found that although morphological, structural, and functional connectivity resemble and interrelate with each other to some extent, there are no determinant even poor correspondences among them.^22^ Furthermore, the neurobiological basis differs among these networks: Morphological connectivity is based on the distribution similarity of regional morphological features; structural connectivity represents physical white‐matter pathways; and functional connectivity reflects statistical interdependences of brain activities. Accordingly, different types of brain networks may differ in their topology and thus reveal complimentary insights into the brain architecture.^12^, ^13^ Consistent with this viewpoint, we found both common and specific topological alterations among the three types of brain networks in patients with anti‐NMDAR encephalitis that were embodied in both global and regional network organization.

The human brain has evolved into an optimal wiring layout, which enables efficient both global and local information transfer to support distributed or integrated and modular or segregated processing. This optimal organization can be quantified by measures of network efficiency at both global and local levels. With these measures, we found decreased local efficiency in the patients in both morphological and structural (FA weighted) brain networks. Local efficiency reflects ability of modular or segregated information processing or fault tolerance of a network and is predominantly associated with short‐range connections. Therefore, the observed decreases in local efficiency imply disrupted segregation in morphological and structural brain networks in anti‐NMDAR encephalitis, presumably due to impaired short‐range connections in the patients. This was supported by our connectivity analyses showing that anti‐NMDAR encephalitis‐related decreases were mainly involved in short‐range connections, in particular for structural brain networks.

We also observed abnormal nodal centrality as characterized by both decreases and increases in the patients. This suggests the co‐existence of pathological impairments and compensatory adaptions in anti‐NMDAR encephalitis.^34^ Specifically, lower nodal efficiency was observed in the amygdala of structural brain networks (FN weighted) in the patients, implying declined efficiency of the amygdala in the coordination with other regions. There is considerable evidence for the involvement of the amygdala in emotion.^35^, ^36^ Given that anti‐NMDAR encephalitis presents various psychiatric symptoms, such as depression,^37^, ^38^ we presume that decreased centrality in the amygdala contributes, to some extent, to emotional dysfunction in the disease. In addition to decreased centrality, we consistently observed the lateral orbital gyrus to show increases among the three types of networks in the patients. This finding highlights the lateral orbital gyrus as a key structure wherein adaptive compensation in brain morphology, structure and function occurs in anti‐NMDAR encephalitis. Previous studies have shown that the lateral orbital gyrus is involved in not only cognitive processing^39^, ^40^ but also autonomic functions via connections with the hypothalamus.^41^ Therefore, increased centrality in the lateral orbital gyrus may be related to autonomic instability in anti‐NMDAR encephalitis.^37^ It should be noted that the lateral orbital gyrus is reported to have strong functional connectivity with the (dorsolateral) amygdala.^42^ It is interesting in the future to explore to what extent pathological impairments in the amygdala are related to compensatory adaptions in the lateral orbital gyrus in anti‐NMDAR encephalitis.

Evidence from autopsy and biopsy showed that patients with anti‐NMDAR encephalitis exhibited extensive microgliosis, moderate inflammatory infiltrates and deposits of IgG that were predominantly involved in the hippocampus, basal forebrain, basal ganglia and cervical spinal cord.^1^, ^43^, ^44^ However, these regions were not detected to show nodal alterations in any type of brain networks. The discrepancy is likely because that the system‐level brain network organization studied here is not sensitive to microscopic histologic alterations in focal regions. It is an interesting topic for future studies to explore the relationships between MRI‐derived brain network findings and histologic postmortem examinations in anti‐NMDAR encephalitis by focusing on specific brain regions relevant to the disease. In addition, several PET studies consistently reported frontal and temporal hypermetabolism and parietal and occipital hypometabolism in the active phase of anti‐NMDAR encephalitis.^45^, ^46^, ^47^, ^48^ Moreover, medial occipital lobe hypometabolism was proposed as an early biomarker for discriminating anti‐NMDAR encephalitis from other encephalitis.^49^ However, abnormalities in these regions were not detected by our network analysis of functional MRI data. One possible reason is that the data of all patients in this study were collected in the recovery phase of anti‐NMDAR encephalitis. Previous studies have shown that abnormal cerebral metabolism can be normalized in the recovery phase of the disease.^45^, ^48^ Given that all patients in this study clinically improved after treatments and that functional brain networks are closely related to cerebral metabolism,^50^, ^51^ it is reasonable to find that these regions were functionally intact in the patients. Further insights can be gained from longitudinal studies by examining therapeutic effects on multimodal brain networks and cerebral metabolism as well as their relationships in patients with anti‐NMDAR encephalitis.

Interestingly, some observed network alterations accounted to a large extent for cognitive impairments of the patients. This finding implies that the network alterations might be neural substrates for cognitive impairments in the patients and suggests related network measures as effective imaging biomarkers to assess and monitor cognitive deficits and therapeutic effects of the disease. Of note, it appears that structural brain network alterations dominated the brain–cognitive relationships in the patients. This observation suggests that white‐matter alterations, such as demyelination and axon injury, may be major factors contributing to pathophysiology of the disease. Moreover, we showed that disrupted structural connectivity exhibited excellent performance for differentiating the patients from HCs. Given that most anti‐NMDAR encephalitis patients showed a normal MRI, these findings are particularly encouraging because network‐based analysis may provide promising ways for objective diagnosis of anti‐NMDAR encephalitis. Notably, the preliminary classification findings were derived from linear discriminant analysis of one‐dimensional features given the relatively small sample size. Future studies should cross‐validate the generalization of our findings by using a large sample size and further improve the classification accuracy by employing sophisticated machine‐learning algorithms that can handle high‐dimensional features (e.g., supporting vector machine).

Several limitations should be addressed in the future. First, the sample size is small in the current study, which challenges the generalizability of our findings to the whole diseased population. Second, a computationally inexpensive deterministic tractography method was used to reconstruct white‐matter tracts due to the huge amount of computation in this study. However, this method has limited capacity in handing complex fiber architectures with non‐uniform fiber orientation, which can be overcome to some extent by probabilistic tractography. Nevertheless, a recent study argues against white‐matter tract reconstruction based on orientation information alone because of systematic false‐positive and negative findings.^52^ Thus, it is warranted to test the reproducibility of our structural network alterations over different particularly newly developed tractography methods. Third, this study investigated the topological organization of multimodal brain networks in patients with anti‐NMDAR encephalitis. It is largely unknown whether other types of encephalitis present similar or dissimilar brain network alterations. It is an interesting and important question for future studies to examine common and specific brain network alterations among different types of encephalitis, and to test whether brain network alterations are able to distinguish different types of encephalitis from each other. Multicenter data sharing will be a practical way for promoting such studies. Fourth, the mRS used in this study is a relative coarse measure for assessing the severity of encephalitis, which may underestimate inter‐patient variability. By utilizing more elaborate measures (e.g., functioning outcome measures), future studies are required to explore the extent to which multimodal brain networks of the patients are affected by different levels of severity of encephalitis. Finally, from a methodological perspective, many factors will affect the topological organization of human brain networks, such as brain parcellation,^53^, ^54^ edge definition,^55^ and thresholding approach.^56^ It is an important topic to identify the most reliable network alterations in anti‐NMDAR encephalitis by examining the effects of different analytical strategies.

## 5 CONCLUSION

The current study provides a comprehensive view of characteristic multimodal network dysfunction in anti‐NMDAR encephalitis, which is crucial to establish new program of auxiliary diagnosis and promising therapeutic targets for the disease.

## DISCLOSURE

The authors claim that there are no conflicts of interest.

## Supporting information

Supplementary Material

## Data Availability

The data are not publicly available due to privacy or ethical restrictions. The data that support the findings of this study are available on request from the corresponding author.
