# Supplementary material for: Aberrant multimodal brain networks in patients with anti‐NMDA receptor encephalitis
Source: CNS Neurosci Ther. 2021 Mar 13;27(6):652–63. doi: 10.1111/cns.13632 (PMC8111502; doi:10.1111/cns.13632)
Supplement: Supplementary file 1 — Supplementary Material [file CNS-27-652-s001.docx]

**Aberrant Multimodal Brain Networks in Patients with Anti-NMDA Receptor Encephalitis**

**Materials and Methods**

**Participants**

We consecutively enrolled 27 patients with anti-NMDAR encephalitis in Xuanwu hospital, Capital Medical University from August 2016 to August 2017. Diagnosis was established based on the characteristic clinical presentation and detection of immunoglobulin G (IgG) NMDAR antibodies. All patients showed positive anti-NMDAR antibodies in the cerebrospinal fluid (CSF) samples according to previously reported criteria ^1,2^. In addition, eleven patients were tested for anti-NMDAR antibodies in the serum and five patients were positive. All patients were diagnosed on admission and received immunotherapy on the same day when the results of antibody tests came out. All patients received first-line immunotherapy (steroids, plasmapheresis or intravenous immunoglobulin), and second-line immunotherapy (cyclophosphamide or rituximab) was given depending on the patients’ status. After 14-day treatment, all patients clinically improved and then underwent MRI scanning. The clinical severity of each patient was evaluated with modified Rankin Scale (mRS) by two experienced neurologists (with 20 years and 6 years of experience in neurology, respectively), and all patients scored from 0 to 2. Thus, the patients included in this study were of favorable clinical outcomes with mild deficits. The control group consisted of 27 age-, sex- and education-matched HCs without any history of neurological or psychiatric disorders that were recruited from local communities via advertisement. All HCs were not tested for anti-NMDAR antibodies in either CSF or serum since it is pathogenic and specific to anti-NMDAR encephalitis. Three patients and one control were excluded from further analysis due to excessive head motion during the functional MRI scan. Among the remaining 50 participants, all 26 HCs and 22 out of 24 patients (91.7%) had a normal brain MRI, as assessed by an experienced neuroradiologist (with more than 10 years of experience in neurology), while the other 2 patients (8.3%) showed non-specific white matter (WM) lesions in the frontal lobe and periventricular areas, as characterized by hyperintensity on T2-weighted images and hypointensity on T1-weighted images. This study was approved by the institutional review board of Xuanwu hospital, Capital Medical University, Beijing, China and written informed consent was obtained from each participant.

**Multimodal MRI Data Acquisition**

All MRI scans (T2-weighted, anatomical, diffusion and resting-state functional) were performed on a 3.0 Tesla MR system (Siemens Magnetom Trio Tim system, Erlangen, Germany) in Xuanwu hospital, Capital Medical University.

The T2-weighted MRI scan was acquired with the following parameters: 35 axial slices; repetition time (TR) = 5,000 ms; echo time (TE) = 87 ms; field of view (FOV) = 256×256 mm^2^; matrix = 256×256; slice thickness = 4 mm; and no gap.

The high-resolution anatomical MRI images were acquired using T1-weighted three-dimensional volumetric magnetization-prepared rapidly acquired gradient-echo (MPRAGE) sequence: 176 sagittal slices; TR = 1600 ms; TE = 2.13 ms; flip angle = 9°; inversion time = 1000 ms; FOV = 256×224 mm^2^; matrix = 256×224; slice thickness = 1 mm; and no gap.

The diffusion-weighted MRI images were acquired using a single-shot echo planar imaging sequence: 80 axial slices; TR = 11,000 ms; TE = 98 ms; flip angle = 90°; FOV = 256×232 mm^2^; matrix = 128×116; slice thickness = 2 mm; no gap; 30 weighted diffusion scans with b = 1000 s/mm^2^ and an unweighted scan (i.e., b = 0); and number of average = 2.

The resting-state functional MRI images were collected using an echo planar imaging sequence: 32 axial slices; TR = 2,000 ms; TE = 30 ms; flip angle = 90°; FOV = 220×220 mm^2^; matrix = 64×64; slice thickness = 3 mm; and slice gap = 1 mm. The scan lasted 6 minutes and 180 volumes were acquired in total for each participant. During the scan, all participants were instructed to keep their eyes closed, relax their minds and remain motionless as much as possible but not to fall asleep.

**Multimodal Brain Network Construction**

In the current study, we constructed large-scale morphological brain networks from anatomical MRI data, structural brain networks from diffusion-weighted MRI data and functional brain networks from resting-state functional MRI data for each participant. A brain network comprises nodes and edges, with nodes representing brain regions and edges representing interregional connectivity, depending on the imaging modality.

***Network Node Definition***. To define brain network nodes, we parceled the brain into 94 regions of interest (ROIs, 47 in each hemisphere) according to the automated anatomical labeling (AAL) atlas in the MNI space ^3,4^. For the structural brain networks, the AAL atlas was warped from the MNI space to individual native diffusion spaces where whole-brain WM fiber tracts were reconstructed. This was done via the inverse transformation of registration from individual T1-weighted images to their b0 images (linear) and then to the MNI space (nonlinear) ^5^.

***Network Edge Definition***. To define brain network edges, we calculated the morphological, structural or functional connectivity between any pair of brain regions as described below.

For the preprocessed anatomical MRI data, the connectivity was estimated using a method similar to previous studies ^6,7^. For each participant, we first extracted GM volume values for all voxels within each ROI. The probability density function of these values was then evaluated at 256 equally-spaced points using a normal kernel function (matlab function, ksdensity), which was further converted to probability distribution function. Subsequently, for any pair of ROIs we calculated the Jensen-Shannon divergence (JSD) between their probability distribution functions, a variation of the Kullback-Leibler divergence (KLD). Compared with the KLD used in our previous study ^7^, the JSD has some notable and useful features, including that it is a symmetric measure and is always a finite value (bounded by 0 and 1 for the base 2 logarithm). Formally, for two probability distribution functions $P$ and $Q$, the KLD and JSD are defined as:

$KLD\left(P||Q\right)=\sum_{i=1}^{n} P(i)\log\frac{P(i)}{Q(i)}$; $JSD\left(P||Q\right)=\frac{1}{2}KLD\left(P||M\right)+\frac{1}{2}KLD\left(Q||M\right)$;

where $M=\frac{1}{2}\left(P+Q\right)$, and n is the number of sample points (256 in the current study). Finally, the morphological connectivity between two regions was defined as the square root of the JSD, followed by a subtraction from 1.

For the preprocessed diffusion-weighted MRI data, whole-brain WM fiber tracts were reconstructed for each participant using a continuous streamline-tracking algorithm ^8^. The tractography was terminated when it reached a voxel with a fractional anisotropy (FA) less than 0.2 or when the turning angle was more than 45˚ between adjacent steps. Two regions or nodes were considered to be structurally connected if there were streamlines with end points located in these two regions. Specifically, we obtained three 94 × 94 structural connectivity matrices for each participant with the elements indicating the number, the mean FA, and the mean length of fiber tracts between regions. These matrices were named FA weighted, fiber number (FN) weighted and fiber length (FL) weighted structural brain networks hereafter.

For the preprocessed resting-state functional MRI data, the mean time series was first extracted for each ROI by averaging the fMRI signals within that ROI. The resultant mean time series were then correlated with each other to generate a 94 × 94 correlation matrix for each participant.

**Graph-based Network Analysis**

In the current study, prior to topological characterization of the multimodal brain networks constructed above, individual networks underwent a thresholding procedure, followed by the calculation of multiple graph-based network measures.

***Thresholding Procedure***. The selection of the thresholding procedure is critical for multimodal brain network studies given the different nature (morphological, structural or functional) and fullness (fully or partially connected) of these networks. Here we restricted individual multimodal brain networks to have the same connectivity backbone as determined by structural networks but allowed variations in connectivity weights depending on the modalities. Thus, all subsequent network calculations were based on weighted networks. Specifically, connections that corresponded to zeros in the structural brain networks were excluded (i.e., set to zeros) for the morphological and functional networks of each participant. This structural network-constrained thresholding approach allows the examination of common and specific network alterations in anti-NMDAR encephalitis under circumstances of fixed connectivity backbone across modalities.

***Network Measure Calculation***. To topologically characterize the thresholded multimodal brain networks, we calculated multiple global and nodal network measures. The global measures included local efficiency ($E_{loc}$), global efficiency ($E_{glob}$) and modularity ($Q$), as well as their normalized versions ($\hat{E}_{loc}$, $\hat{E}_{glob}$ and $\hat{Q}$) by matched random networks (n = 100). The random networks were generated using a random rewiring algorithm to ensure the same number of nodes and edges and the same degree distribution as the real brain networks ^9,10^. At the nodal level, we calculated five nodal centrality measures, including the nodal degree ($k_{i}$), nodal efficiency ($e_{i}$), nodal betweenness ($b_{i}$), nodal eigenvector ($v_{i}$) and nodal pagerank ($p_{i}$). The calculation, use and interpretation of these global and nodal network measures can be found in previous studies ^11-13^.

**Statistical Analysis**

In this section, we compared between-group differences in demographic, cognitive and multimodal network measures. For network measures showing significant alterations in the patients, we further examined their associations with clinical and neuropsychological variables of the patients and their discriminant power in distinguishing the patients from HCs.

***Between-group Differences***. Discrete sex data were analyzed using a chi-square test. For other demographic and cognitive continuous variables (age, education, MMSE, SDMT, CVLT, BVMT and PASAT), two-sided two-sample t tests were used to infer between-group differences.

For network measures (global and nodal) and cross-modality relationships, between-group differences were statistically tested using nonparametric permutation tests. Brieﬂy, for each variable, we first calculated the between-group difference in their mean values. An empirical distribution of the difference was then obtained by randomly reallocating all values into two groups and recalculating the difference in mean values between the two randomized groups (10,000 permutations). The 95th percentile points of the empirical distribution were used as critical values in a one-tailed test of whether the observed group differences could occur by chance. For all permutation tests, effects of age, sex and education were controlled by treating them as covariates. Additionally, the mean frame-wise displacement of head motion was treated as an extra covariate for comparisons involving functional brain networks. For each type of brain networks, a Bonferroni method was used to correct for multiple comparisons for global network measures (*P* < 0.05/6 = 0.008), and a false discovery rate (FDR) procedure was used to correct for multiple comparisons for nodal network measures at a q value of 0.05 (470 comparisons). In addition, the FDR procedure was used to correct for multiple comparisons for 60 cross-modality comparisons.

To examine between-group differences in interregional connectivity, a network-based statistic (NBS) method ^14^ was used. Briefly, for each modality (morphological, FA weighted structural, FN weighted structural, FL weighted structural and functional), a *t*-value matrix (94 × 94) was first derived by an edge-by-edge between-group comparison of interregional connectivity strength (two-sample *t*-test). A primary significance threshold (*P* < 0.05) was then applied to the *t*-value matrix to select suprathreshold connections, among which all connected components were identified and their sizes (i.e., number of links) were recorded. To estimate the significance of each identified component, a null distribution of the connected component size was empirically derived using a permutation approach (10,000 permutations). For each permutation, all participants were randomly rearranged into two groups, and the same primary significance threshold (i.e., *P* < 0.05) was used to filter suprathreshold links for the edgewise comparisons between the two randomized groups. The size of the maximal connected component among these links was recorded to form the null distribution. Finally, for any connected component of size M that was observed in the comparison of the right grouping, the corrected *P* value was determined by calculating the proportion of the 10,000 permutations for which the maximal connected component was larger than M. Again, effects of age, sex and education were controlled for comparisons of each modality, and the mean frame-wise displacement of head motion was added as an extra covariate for functional comparisons. Notably, for structural brain networks (FA weighted, FN weighted and FL weighted), only connections that existed in > 60% of all participants were included in the NBS analysis.

***Relationships between Multimodal Brain Networks and Clinical/Neuropsychological Variables.*** For multimodal brain network measures that showed anti-NMDAR encephalitis related alterations, Pearson or Spearman correlation analyses were used to examine their associations with the clinical and neuropsychological variables of the patients (disease duration, MMSE, SDMT, CVLT, BVMT and PASAT 2 and PASAT 3). The FDR procedure was again used to correct for multiple comparisons (70 correlations).

***Receiver Operating Characteristic Curve*.** To examine whether the multimodal brain network measures might serve as potential biomarkers for differentiating the patients with anti-NMDAR encephalitis from HCs, the receiver operating characteristic (ROC) curve was plotted for each measure that showed significant alterations in the patients. Specifically, for each network measure or feature, many different thresholds were used to classify each participant into either patient or control group. For each threshold, the fraction of correctly identified patients (i.e., sensitivity or true positive rate) and the fraction of correctly identified controls (i.e., specificity or true negative rate) were calculated. Finally, a cut-off point that simultaneously optimized the sensitivity and specificity was determined and the classification accuracy at the cut-off point was calculated as the fraction of correctly identified participants (both controls and patients). This procedure was performed using the public MATLAB codes (http://www.mathworks.cn/matlabcentral/fileexchange/19950-rocout=roc-varargin-; Giuseppe Cardillo, Naples, Italy). For measures showing good (area under curve > 0.8) or excellent (area under curve > 0.9) discriminative performances, we further determined whether the discriminative performances could occur by chance (permutation test) and were robust against small data fluctuations (bootstrap procedure) ^15^. First, for each measure, all the participants were randomly reallocated into two groups, based on which the ROC analysis was conducted to generate an empirical distribution for the area under curve (10,000 permutations). This distribution was used to estimate the significance level of the observed discriminative performance. To further examine the robustness of the discriminative performance against small sample size, we generated 10,000 bootstrap samples for each metric by resampling without replacement, and each bootstrap sample included 19 anti-NMDAR encephalitis patients and 21 HCs (i.e., 80% of each group). The ROC analysis was then performed for each bootstrap sample to obtain an area under curve distribution, based on which the 95th percentile points were used to determine whether the observed discriminative performance fell within the 95% confidence interval of those derived from the bootstrap samples. Finally, the FDR procedure was used to correct for multiple comparisons.

**Post-hoc Examination of Susceptibility Artifact Signal Loss**

To examine the susceptibility artifact signal loss in the right lateral orbital gyrus, a region that was consistently found to show increased centrality in the patients, we performed the following analyses. Specifically, for both the diffusion and functional MRI data, we separately extracted 1 volume (the tenth) from each participant’s time series ^16^ and compared the mean signals (across voxels) in the right lateral orbital gyrus between the two groups (permutation test, 10,000 permutations). If significant between-group differences were observed in the signals, the corresponding values were treated as an additional covariate for between-group comparison of nodal centrality of the right lateral orbital gyrus.

**References**

1. Jones KC, Benseler SM, Moharir M. Anti-NMDA Receptor Encephalitis. Neuroimaging Clin N Am. 2013;23(2):309-320.

2. Dalmau J, Lancaster E, Martinez-Hernandez E, Rosenfeld MR, Balice-Gordon R. Clinical experience and laboratory investigations in patients with anti-NMDAR encephalitis. Lancet Neurol. 2011;10(1):63-74.

3. Tzourio-Mazoyer N, Landeau B, Papathanassiou D, et al. Automated anatomical labeling of activations in SPM using a macroscopic anatomical parcellation of the MNI MRI single-subject brain. NeuroImage. 2002;15(1):273-289.

4. Rolls ET, Joliot M, Tzourio-Mazoyer N. Implementation of a new parcellation of the orbitofrontal cortex in the automated anatomical labeling atlas. NeuroImage. 2015;122:1-5.

5. Gong G, He Y, Concha L, et al. Mapping Anatomical Connectivity Patterns of Human Cerebral Cortex Using In Vivo Diffusion Tensor Imaging Tractography. Cereb Cortex. 2009;19(3):524-536.

6. Kong XZ, Liu Z, Huang L, et al. Mapping Individual Brain Networks Using Statistical Similarity in Regional Morphology from MRI. PloS one. 2015;10(11):e0141840.

7. Wang H, Jin X, Zhang Y, Wang J. Single-subject morphological brain networks: connectivity mapping, topological characterization and test–retest reliability. Brain and Behavior. 2016:n/a-n/a.

8. Mori S, Crain BJ, Chacko VP, van Zijl PC. Three-dimensional tracking of axonal projections in the brain by magnetic resonance imaging. Ann Neurol. 1999;45(2):265-269.

9. Maslov S, Sneppen K. Specificity and stability in topology of protein networks. Science. 2002;296(5569):910-913.

10. Sporns O, Zwi JD. The small world of the cerebral cortex. Neuroinformatics. 2004;2(2):145-162.

11. Rubinov M, Sporns O. Complex network measures of brain connectivity: uses and interpretations. NeuroImage. 2010;52(3):1059-1069.

12. Wang JH, Zuo XN, Gohel S, Milham MP, Biswal BB, He Y. Graph theoretical analysis of functional brain networks: test-retest evaluation on short- and long-term resting-state functional MRI data. PloS one. 2011;6(7):e21976.

13. Zuo XN, Ehmke R, Mennes M, et al. Network centrality in the human functional connectome. Cereb Cortex. 2012;22(8):1862-1875.

14. Zalesky A, Fornito A, Bullmore ET. Network-based statistic: identifying differences in brain networks. NeuroImage. 2010;53(4):1197-1207.

15. Shen Y, Yao J, Jiang X, et al. Sub-hubs of baseline functional brain networks are related to early improvement following two-week pharmacological therapy for major depressive disorder. Hum Brain Mapp. 2015;36(8):2915-2927.

16. Greicius MD, Flores BH, Menon V, et al. Resting-state functional connectivity in major depression: abnormally increased contributions from subgenual cingulate cortex and thalamus. Biological psychiatry. 2007;62(5):429-437.
